# Pneumonia Acute—Its Treatment, Etc.

**Published:** 1873-07

**Authors:** A. S. Payne

**Affiliations:** Virginia


### SOUTHERN MEDICAL RECORD.

VOL. III.—JULY, 1873.—No. 7.

### PART I.

### ORIGINAL COMMUNICATIONS.

### PNEUMONIA ACUTE—ITS TREATMENT, ETC.

BY A. S. PAYNE, M.D., OF VIRGINIA.

In speaking of pneumonia, I have determined to offer a few suggestions, intended more especially for the benefit of the younger members of the medical profession. The thought has occurred to my mind more than once, that the *florid* importance given to the subject of "Inflammation" by the professors in some of our medical colleges is more productive of harm than of go[illegible]d. Inflammation eloquently pictured by these learned professors makes an impression upon their young and sensitive imaginations that haunts them as a night-mare during the remaining period of their natural lives. The great majority of men in and out of our profession are by nature "timid." With such men, however good their intellectual endowments may otherwise be, early impressions on the *scarry* side of any question become with them very enduring. Indeed, it many times appears to rather "grow with their growth and strengthen with their strength." I have seen such timid practitioners giving one grain of sulphate quinine every three or four hours, in diseases of a very decided asthenic type, and this one grain of quinine hampered by combining with it one-fourth of a grain of pulvis ipecac, or an infusion of eupatorium perfoliatum—their timidity being so great from early impressions that they were actually afraid to "blow hot" even in such a minute dose without "blowing correspondingly cold" at the same time—when five to ten grains of quinine and three to five grains of pulvis doveri would hardly be sufficient to sustain the system of their patient. But ask them why they did not exhibit the remedy in larger doses, and they will invariably tell you they feared a "determination of blood to the brain," or "inflammation of the bowels," kidney or bladder. They entirely ignore the patent fact, that too little blood circulating through the brain and its appendages in a given number of seconds as surely produces delirium as does too much blood circulating through the same parts in a given time, the "delirium" only differing in character—too much blood thrown with increased force producing a fierce, active delirium, as in congestions of the meninges of the brain, whilst too little blood circulating with enfeebled force gives rise to a low, muttering, wandering sort of delirium, such as is witnessed in typhoid fever—the first to be controlled, moderated by depletants, the latter equally benefitted by stimulants, to be exhibited in full doses. It will not surprise you when I tell you that with such timid practitioners that in every irritation, etc., they see the ghost of an inflammation turning up in huge proportions before them. For the man that goes to the graveyard especially to see a ghost will rarely fail to see one. I am very glad to know that very able men (assisted by microscopical investigations) have taken in hand this important and interesting subject, and to-day it is a closely disputed point whether inflammation is the consequence of plus vitality or of minus vitality. Whilst I am of the opinion that inflammation may be the result of either plus vitality or of minus vitality, yet, the learned discussion can not fail to do good, if only in strengthening the "weak-kneed" of our profession. I was once thrown in contact with a young physician (occupying the same office) of good mind and fine advantages, who, nevertheless, came from Philadelphia with the "peritoneum on his brain." Every time he returned from visiting a patient who had any trouble about the bowels, he would expatiate to me on the beauties of his case of peritonitis. If a case of midwifery, on his return from his second visit he was sure to tell me of some symptoms of threatening puerperal peritonitis which he foresaw in the distance. This continued for several years, and I verily believe he would be seeing cases of peritonitis yet, but he talked so much about the peritonium and his cases of "peritonitis," that finally the boys took it up, and would sing out when they saw him passing the street, "there comes old peritonitis." This M.D., although he is an old practitioner, and has seen as much practice as most physicians, is still very prone to scent inflammation afar off, and in consultation cautions you about the propriety of exhibiting stimulants and sedatives, it matters not how strongly the asthenic type marks the case. Particularly is this so if any symptom of delirium is apparent; he draws no distinction between the symptoms of the deilrium, whether it is fierce and active, or low, muttering and wandering in its character. Generally the most fearless men are the most vascillating and timid in the practice of their profession. In fact, they are the victims of early impressions moulded upon their youthful minds in the lecture-room by some eloquent and favorite professor. Gentlemen, I advise you always to do your own thinking, and never, never take any thing for granted. Examine every case, every thing connected with the case critically for yourself. Then in every case ask yourself this question: Does Nature really need any assistance at my hands to enable her to throw off this disease? If she does not, or you are in doubt, administer some pleasant placebo and closely watch her efforts; for if she does not require your assistance, you will rather bother, hamper her in her laudable efforts to eradicate disease than be of any benefit to her. Her efforts are always exerted in the interest of health against disease, and all she needs is a "helping hand" given in the right direction and at the proper time. If on the other hand you are "sure you are right," and that Nature must have your assistance, and what that assistance must be, then "go ahead;" let your assistance be as simple, as uncomplicated as possible, but let it be prompt, decided, sufficient. Don't procrastinate needlessly, nor *higgle* over the size of the dose; be clear as to the effect you wish to produce, and be sure to give the dose large enough to produce it. If you do otherwise, you are at best only trifling with the remedy, and with the disease at the same time. To illustrate the importance of examining every case for yourself,

I will relate a "little case" that actually occurred in my neighborhood when I was a much younger man than I find myself to be to-day. I remember that this case caused a profound sensation in the neighborhood at the time, for the attending physician was a good talker, and much given to relating his cases to his neighbors. Dr. L. was called to see a little child of J. R.'s, two or three years of age. He found the child lying on its back, feverish, moaning and continually tossing its left arm over its head. Always on the "*qui vive*" for inflammation of the brain or its coverings. The moaning and tossing of its arm over its head was quite sufficient to induce the Doctor to believe he had a very interesting case of "cerebrites" to treat. Thus believing, he diagnosed inflammation of the cerebrum, and he ordered the child to be kept in a dark room, still, not to be disturbed even by undressing, with cloths dipped in ice-water to be kept continually to its head, etc. After practicing three days without any change, the Doctor summoned Dr. ——, from afar off, for consultation on the interesting and important case of inflammation of the cerebrum. The second Doctor gave, after seeing the child and noticing some fancied weakness about the urino-genital organs, as "his opinion that the cerebellum was the seat of the disease." At this juncture, about the seventh day of the case, I was called by the father of the child. When I arrived he said: "Dr. P., you are a stranger to me; Mr. S. says you have some common sense; will you examine my child and tell me in plain language what you think of it?" "Certainly, sir." I determined to examine the child from the crown of its head to the soles of its feet. In stripping off its clothes to do so, a pen-knife dropped out from the axilla of its left arm that had become wedged in by a tight sleeve. The excoriation of this region by the knife was the cause of the tossing of its arm over its head, and of the moaning—the child not being allowed to be undressed by the mother, was the cause of the knife not being discovered sooner. This interesting case of cerebritis was thus brought to a speedy and a happy end. Were such a case to occur now-a-days, we know it would rejoice under the cognomen of cerebro-spinal meningitis. For fashion holds potent sway even in disease. In treating a disease there are two important points to notice: First, the "tendency to death;" this must be met by the proper remedies to evert this tendency. Second, the indications of cure required in each case. In pneumonia, one of the most important (if not the most important) is to procure sleep, to promote the arterialization of the blood, and to empty the pulmonary blood vessels of their engorgements. Sleep is best procured by large doses of nerve-stimulants, ergo sedatives. The remedies producing nerve-force are always soporific, given in full doses. I have seen as profound sleep produced by large doses of quinine as I ever did by the administration of opium. The engorgement of the pulmonarry vessels is best gotten rid of by a large blister applied over the branches of the great par vagum. This never transmits its stimulation to the heart, which contracts more powerfully upon its contents, and a freer current of blood is thrown out through the vessels of the lungs, (so to speak), which washes away the stagnating fluid, and your engorgement is relieved. The engorgements about the portal system is most successfully overcome by the administration of bilious purgatives, of which sub. mur. hydr. and the compound ext. of colocynth are the best. The great desideratum in pneumonia, sleep, without which all treatment will prove of no avail, must be produced by large doses of quinine, or by a combination of quinine and pulvis doveri, morphia or ext. aconite.

Many cases of so-called acute pneumonia are simply a stasis of blood in the lungs. Others are entitled to the more dignified appellation of engorgement of the pulmonary vessels; and other cases again, of so much severity that they may, with propriety, be termed "apoplexy of the lungs." They all, however, with rare exceptions, owe their origin to a predisposing cause furnished by the system, and an exciting cause furnished by atmospheric alternations—malaria; a malaria very similar to that at other times producing remittent, bilious and intermittent fevers. Although long suspected by others, Dr. R. LaRoche was the first boldly to assert this doctrine. Inflammation is recognized by heat, redness, swelling. Then I contend that in acute pneumonia the first departure from a physiological state to a pathological condition is not inflammation of the parenchymatous structure of the lungs, but rather a stasis of blood, an engorgement of the blood vessels; and if the accumulation in the vessels is great, it amounts to apoplexy of the lungs. These abnormal conditions, if not reduced by the appropriate remedies and applications, may result in inflammation, suppuration or gangrene. I am in the habit of satisfying myself by actual experience, acquired at the bedside of the patient, as to the most available treatment to be adopted in a given disease. Fully satisfied upon this point, I then look around me for a theory that most reasonably "squares" with my treatment. I do not make my treatment of a disease square to any theory of it, but rather build my theory to "square" to my treatment. Had I found in practice venesection, antimonials, etc., to be the best remedial treatment for acute pneumonia, I had never been led by investigation to come to the conclusion that oftentimes the disease is rather a stasis of blood than an inflammation of the parenchymatous structure of the lung.

I shall, therefore, attempt to establish in this paper that venesection, the exhibition of antimonials, expectorants, mercurials, etc., is not the best plan of treatment for the so-called acute pneumonia. In inflammation of the serous membranes, venesection is always beneficial. In irritations and inflammations of the mucous membranes, if ever beneficial at all, it is so as a mechanical remedy only. I will submit the question: If a large amount of poor blood can not support the system, how can a smaller amount of the same furnish any better support? By the application of your lancet, you do not propose to improve the quality, but only to lessen the quantity.

To more fully illustrate my peculiar notions of so-called acute pneumonia, pulmonary apoplexy, etc., I shall select a few cases of each that have occurred in my own practice in Piedmont-Virginia, running through a period of twenty-seven years. In all the cases I shall mention, the patients are alive now, and hardy and able to speak for themselves, if necessary; moreover, I assert, not a symptom or incident is recorded here but is meant in all seriousness, and actually occurred. I make this remark because in a few of these cases the treatment is so novel, and at first sight may be deemed harsh, until we reflect; the recoveries remarkable, until we remember the tenacity of life with some individuals.

But before I go further, the reader may naturally want to know how I diagnose a case of so-called acute pneumonia. What are its characteristic symptoms? How do you distinguish the so-called acute pneumonia from inflammations of the serous membranes?

from pleurisy? from acute bronchitis? from typhoid pneumonia? from acute phthisis? from bilious pneumonia? from pulmonary œdemia, etc.? I will try to answer these questions to the best of my poor ability.

You may suspect acute pneumonia when the breathing is very rapid, the fever severe, and if, in addition to râles, not very well defined spots of dullness, which do not change their seat, be discovered. On the other hand, when there are symptoms of deficient arterialization or aeration of the blood; when the symptoms point more to prostration than to activity of febrile symptoms; when the patient seems to suffocate from want of power to expectorate; when multitudes of fine, dry râles are heard at every part of the chest, and little or no correspondiug impairment of the natural resonance is detected on percussion. The hot, dry skin, the flushed face, the quickened pulse, the rapid breathing, the thoracic pain, the cough and the peculiar expectoration point out at once the nature of the attack, and the organ which is disturbed. The decubitus is usually dorsal, with legs drawn up. Commonly beginning with a chill, or with flashes of heat, the disease progresses with the symptoms indicated. The expectoration is very characteristic; it consists at first of a dry, glassy mucus; soon it becomes more viscid, and acquires that significant appearance dependent upon the admixture of blood with the mucus and exudation matter to which the term "rust-colored" has been given. The rusty-colored sputa has been considered by many authors pathognomonic of acute pneumonia; yet it will be well to be aware, says Dr. Watson, that cases of pneumonia run their course without it. The expectoration may be purulent, or it may be like "prune juice"—according to my experience, a symptom characterizing only very serious attacks of pneumonia. The cough may, in pneumonia, also be said to be peculiar, being more of an effort to cough than "coughing." Another marked symptom is the shortness or increased frequency of breathing. The patient draws from forty to eighty breaths a minute, but the pulse, although rapid, does not quicken in proportion. Pneumonia, therefore, forms an exception to the rule that with greater frequency of breathing the pulse rises.

The febrile symptoms are ordinarily very severe; still they are not associated with decided cerebral disturbance. Headache is common: delirium rare. The heat of the skin is burning, and the flush on the cheek so decided that, by this and the hurried breathing alone, the disease may often be recognized. The flush is said, by Bouillard, to be more obvious and of a darker color, when the apex of the lung is affected. The urine is high colored. The nitrate of silver fails to precipitate its chlorides. Dr. Walshe considers this an important element of diagnosis.

In addition, authors give these additional physical signs of acute pneumonia: In the first stage of engorgement occur increased vascularity and commencing exudation in the air-cells, into which, however, the air is still capable of entering. The vesicular murmur is somewhat altered; it may be fuller or longer. But soon are heard, with each act of inspiration, numerous equally and rapidly evolved very fine, crackling sounds, the crepitant râles of Laennec. As the exudation becomes firmer, the tissues of the lung solidifies by occlusion of the air-cells, and the stage of red hepatization (of authors) is before us. Now, all the signs of complete hepatization are discovered. We find decided dullness on percussion; blowing respiration in all its purity; high-pitched and tubular sounding; bronchophony and increased vocal fremitus. Râles from the accompanying bronchitis are heard with extreme distinctness through the solidified tissue, (Skoda's consonant râles), and so are the sounds of the heart. A crepitant râle is here and there perceptible, or the ear catches a friction sound. When the exudation is reabsorbed or expectorated, the signs of consolidation become less and less perfect; a vesicular bronchical respiration succeeds the bronchical breathing. The dullness on percussion lessens; crepitant râles, not, however, so fine as at the outset of the affection, and, mixed with larger moist râles, return. The cough increases, the expectoration becomes more copious, thickens, loses its tenacity, its rusty color, and is expectorated in "thick chunks." The dyspnœa diminishes rapidly, indicating a "breaking up" of the exudation and return of air into the cells. If, instead, the exudation be converted, externally, into pus and the lungs soften, the physical signs are the same as in the second stage. The rarity of excavations of sufficient size, explains why gurgling and the sounds of a cavity are not perceived. We suspect the mischief that is going on within the cavity from the protracted dyspnœa, the increasing rapidity of the pulse, purulent or brownish sputa, pinched features, the dry tongue and the mental wandering. This is the third stage—the gray hepatization (of authors).

The first is the stage of engorgement (stasis) and the commencing exudation, crepitant râle, slight dullness on percussion, cough, beginning dyspnœa and fever. Second, the stage of solidification of lung tissue, (red hepatization) progressive dullness, bronchial respiration, bronchophony, rusty colored sputum, dyspnœa, cough, high fever, absence of chlorides in the urine. Third, stage of softening (gray hepatization) the same as is second stage, unless large abscesses have formed, chills, prostration, etc., purulent or brownish sputum.

Here, then, we have a disease which presents such striking symptoms and signs in all its phases, in which the sputum are so peculiar, the hurried breathing so evident, the physical signs so distinct, that error is, with an ordinary case, difficult. It becomes still more so, if a few of the pathological peculiarities of pneumonia be borne in mind, that it comparatively seldom effects the upper lobes of the lungs, and that it is often accompanied by the signs of slight bronchitis or pleurisy. Again, the feverish pulse ratio of Dr. Walshe may be made an important element in the diagnosis.

In pleurisy, the pain is sharp; you have dry cough, impaired chest motion "to and fro," or friction sound. In the second stage of pleurisy, you have obliteration of the intercostal spaces, enlargement of the side, displacement of viscera. In a large majority of cases, dullness, with enfeebled or absent respiration, voice and fremitus; decubitus on the affected side; sputa frothy; rarely any râles in the chest. The friction sound I deem pathognomonic of the fact that the disease has invaded the pleura.

Typhoid pneumonia is pneumonia with primary symptoms of a typhoid or asthenic type, and marked by rapid failing of the vital powers. The crepitant râle is less frequent, less abundant. There is, however, such manifest prostration attending typhoid, both mental and physical, from its inception, that acute pneumonia could hardly be confounded with it.

Bilious pneumonia is pneumonia, sthenic or asthenic, wearing the livery of malaria. Bilious pneumonia is regarded by some writers as nothing more than a special form of remittent or intermittent fever, in which the lungs are made to bear the burden of the disease. It is held by others to be a variety of pneumonia occasioned by the ordinary causes of pneumonia, but owing its peculiar symptoms to its happening in those in whose systems the poison of malaria has been slumbering.

In regard to acute phthisis, it is only necessary to remark that cases are encountered, apparently of pneumonia, in which, after the symptoms of acute pneumonia pass off, those of phthisis come into the foreground.

In regard to acute bronchitis, I will merely recall that the dyspnœa is not so great, and that no dullness is yielded on percussion by an inflamed bronchial membrane. Here, percussion is of signal value in the diagnosis of pneumonia. In fact, when acute bronchitis complicates pneumonia, and loud, dry râles take the place of the blowing respiration, it is our only trustworthy guide. A simple tap on the chest, which elicits an absolutely dull sound, tells the difference between pure bronchitis and inflammation (or, as I think, engorgement) of the bronchial mucous membrane accompanying inflammation (engorgement) of the parenchymatous structure of the lung. There is, however, no doubt that pneumonia is sometimes mistaken for œdema, pulmonary apoplexy, pulmonary engorgements during attacks of fever. These facts strongly militate in favor of the theory I have the honor of submitting to my professional brethren, that pneumonia is not generally an imflammation of the parenchymatous structure of the lungs, but an engorgement, or rather, primarily, a stasis of blood in these parts—owing its origin to climatic alternations, capable of producing a malaria alike in the mountain and the lagoon, but nevertheless a malaria—a blood poisoner.

In my views of the pathological condition we usually find in the inception of an attack of acute pneumonia, as well as with the best plan to be adopted for its treatment, I differ so much with my professional brethren, that I think it not amiss to give a short sketch of the plan mostly adopted by the general practitioner in my section of the State, before I place upon record a few cases of pulmonary apoplexy, pulmonary engorgement, and the so-called acute pneumonia, as illustrative of my own plan of treatment for these several degrees of lung congestions. I shall only mention briefly a few very striking cases of each variety of lung congestions, although I have encountered many in my practice in this region of country, running (as I before said) through a period of twenty-seven years.

The practice usually adopted by the profession in this section of the State is that of the great Sir Thos. Watson, somewhat modified, it is true, by the individual notions of the practitioner, but nevertheless, in the aggregate, is the practice of Dr. Watson. And here we should remember, that the great majority of cases seen by Dr. Watson were Englishmen, a heavy feeding, beef-eating, porter-drinking race, whose systems are generally surcharged with rich fibrinous blood, abounding in red blood corpuscles—a class of patients differing widely from those we are usually called upon to treat in the more mountainous regions of Virginia. Dr. Watson, remarkable for his acumen and segacity, says: "The great instruments to be employed in the treatment of inflammation of the lungs, are the same which have so often been recommended by me in other inflammatory affections before: blood-letting, tartarized antimony, mercury. Of these, blood-letting is the chief." "Both reason and experience attests the especial power of bleeding upon acute pneumonia." In the first place, it tends to restrain or extinguish the inflammation." But in the next place, it has the effect of relieving the particular functions of the lungs." "The late Dr. Gregory, of Edinburgh, was in the habit of saying in his lectures, that provided he was called early to a case of pneumonia, he would be content to dispense with all other aids, than those of a lancet and water gruel." "I am far from desiring you to believe that blood-letting is the only expedient required; but certainly, the amount of the best experience, ancient and modern, is strongly in favor of its free, and I might almost say, its prodigal employment." "Very lately one most distinguished French writer, M. Louis, has endeavored to show that venesection has not much control over the progess or issue of pneumonia." "And I advert to his opinion on this subject, merely to caution you against being misled by it, as you might otherwise be, considering his well-merited reputation as an exact and faithful observer." Here, although Dr. Watson finally commits himself to the doctrine of abstraction of blood in pneumonia, still, there is a vascillation, a blowing of hot and cold not usual with this great author. He is not firm in his opinion, he, evidently, has his suspicions. But let us follow this candid, sagacious man a little further. Hear him. "But a time arrives when bleeding is no longer of use, or when it is positively hurtful, when it ceases to have any good influence on the local disease, and has an injurious influence on the *whole* system, reducing the patient's strength, and incapacitating him from 'bringing up' and ridding his lungs of the tenacious mucus exhaled by the bronchial membrane." This is what takes place in those cases in which the expectoration is said to be stopped by a bleeding." (Just what I have seen occur from venesection in the inception of a case of so-called acute pneumonia.) "I have mentioned Dr. Gregory's reliance on blood-letting for the cure of pneumonia; and I ought to tell you at the same time (I think so too) what I have been informed respecting the result of his practice." "He used to bleed to the verge of convulsions." "His colleague, Dr. Rutherford, seldom went beyond three bleedings, and generally accomplished his object by two, judiciously timed and measured." "His patients recovered quickly; Dr. Gregory's very slowly."

Here you see a little light creeping through the cracks of this beautiful compilation. But let us go on. "All that I have been hitherto saying, relates to acute pneumonia, occurring in a previously healthy person." Hale, hearty, beef-eating, porter-drinking John Bull. "But pneumonia having that character, and so occurring, is a much less common disorder than most persons appear to suppose, or than I formerly thought." "I have been surprised to find how few cases of pure idiopathic inflammation of the lungs present themselves among my hospital patients." So, you perceive, he is gradually coming over. He admits that those patients bled most recover the slowest, and, finally, that pneumonia requiring the lancet is a rare disease, much more so than he himself formerly supposed. I hereby submit, with all due respect for these two celebrated physicians, that my patients in this mountainous region, get well quicker than either, without any blood-letting at all.

On the 25th day of October, 1855, I was called to see Jerry Munday, a stout colored man, twenty-six years of age. Jerry was seen by my friend Dr. G. M. B—— on the morning of the 23d, and again on the morning of the 24th. The Doctor did not consider Jerry much sick, and at both visits prescribed blue mass grs.

x., to be followed each time by a tablespoonful of castor oil. On the morning of the 25th, Dr. B—— and myself received a hasty summons from Mrs. R——, his mistress, to visit Jerry. I arrived a few moments ahead of the Doctor. Jerry being supposed to be in a dying condition by his fellow-servants, Mrs. R—— insisted upon my going in at once to see him. This I did not hesitate to do, as the Doctor was one of my most intimate friends. The following was the condition I found him in: Lying on his back; extremities cold; bathed in a cold, clammy sweat; pulse very rapid, but weak; nearly speechless; semi-comatose; countenance anxious, pinched and shrunken; inability to expectorate. I could see upon the wall around his bed the peculiar, tenacious, rusty-colored sputa that he had spit up the day and night before. All his symptoms were very unpromising. I had hardly returned to the house before the footsteps of Dr. B—— were heard in the porch. We quickly returned to see the patient together. After an examination, Dr. B—— gave it as his opinion that he would not live beyond sundown. I made the following proposition to the Doctor, which he frankly accepted: "Dr. B——," said I, "you know we differ somewhat in our treatment of pneumonia, and you admit that by the old mode of giving tartarized antimony, expectorants, etc., he has but a slim chance for his life. Suppose, now, you permit me to take my own plan of treatment with this case." Dr. B—— replied: "I have no objection in the world; but in selecting this case, I do not think you are doing your plan or yourself justice." But knowing that he had youth on his side; that the previous treatment was very favorable, as the portal system had been thoroughly unloaded by the mercurials and the oil; and that, if successful, (which I did not for a moment doubt), it would approach somewhat to the marvelous in the eyes of the Doctor himself and the attendants, I therefore gladly, fearlessly took sole charge of Jerry. I apply to his whole chest a fly blister, to remain on until well drawn; give him thirty grains of sulphate quinine, thirty grains to be given at sundown, and thirty grains at sunrise next morning; buttermilk, half water, *ad libitum*, or beef tea—Dr. B—— and myself to meet at Mrs. R.'s at breakfast next morning. Met promptly—learned that Jerry was decidedly better. After finishing our breakfast, go to the cabin to see him; find

Jerry sitting up on the edge of his bed; whole appearance is changed; blister has drawn well; pulse eighty, full; expectoration copious and easy—comes up in thick chunks; Jerry is convalescent. Directed beef tea, buttermilk half water, or nice soup with the top skimmed off, *ad libitum*, with a little warm toddy occasionally; to be continued for a few days. No relapse. Have lost sight of this patient since the war.

Was called March 13th, 1854, arriving at three P.M., to see O. C——, a good, clever man, of nervo-bilious temperament, good-natured enough, but terrible in his wrath when raised. He has attempted to plow a rough, hilly, stony piece of ground with a "balky" horse. He has overheated, perfectly exhausted his muscular system by fighting this horse, and his mental system has become perverted by cerebral congestion superinduced by terrible passion. He is perfectly crazy, requiring four men to hold him in bed. His pupils are contracted, his countenance fierce, face pale; pulse scarcely larger than a thread, very rapid, too rapid to count, and wiry; with difficulty spits up occasionally a glassy, "rust-colored" sputum; skin hot, dry, pungent. Whole appearance is such that I think he has had a hemorrhage from his bowels; can not ascertain this fact. With much trouble get a fly-blister over the whole chest, and give quinine grains thirty. In a few minutes he fixes the pillows under his head and lies down; he only lies quiet for a few moments; he has to be held again, but is held with *much* less difficulty. At four P.M. I gave twenty grains more of quinine; in five minutes thereafter he is profoundly asleep. To prevent his being disturbed, by attendants purposely waking him, which will cause his death, or at least insure his being sent to Staunton, I remained with him all night. It is well that I did so, for I had hard work to prevent his mother-in-law and another old lady from waking him up during the night. I would pretend to be sleeping, but just as they would be about to shake him, I would rouse up in time to put a stop to their intentions. During the night I heard the mother-in-law whisper to the other old lady, "He'll never wake; he's fixed him; he has given him too much 'morphy.'" Precisely at eight A.M. the next morning he awoke, asked for a drink of water, and drank heartily. Blister has drawn well; expectorates easily; expectoration has thickened; is himself; is composed, convalescent. Advised a few days' rest, light soup diet, with a little warm toddy every few hours. No relapse.

In 1857 I saw him in a very similar situation from taking a severe cold. Sixty grains of quinine given him as before, promptly relieved him. He is alive, well, and my nearest neighbor.

October 10th, 1835, I was called to see Whiting, H., about forty-five years of age, a man with no surplus flesh, and naturally of a pale, cadaverous caste of countenance. I found on my arrival, late in the day, that Dr. B., of Clark county, had been sent for also, and the Doctor was on his horse just in the act of leaving when I arrived. I urged upon him to get down, but he persistently refused to do so, saying the patient was dying and he had the Shenandoah river to cross, and wished to cross before it was dark. Left to my own resources, I surveyed the ground, determined to make the best fight I could with the disease. His attack was brought on by, his wife informed me, over-exertion, fatigue and passion, as he had, also, attempted to plow a piece of ground with "balky" horses. I must confess he had all the appearance of one in a fatal stage of collapse. I found him semi-comatose; pulse scarcely perceptible at the wrist; surface of the body cold; countenance pale, cadaverous, anxious; breathing laborious, humid, decubitus dorsal; unable to cough, much less to expectorate, although you could see where he had previously expectorated a "rusty, brown colored" sputa; his bed was found to contain a large quantity of thin serous-looking blood, passed per ano. I "locked up" his bowels with an enema composed of water, starch and laudanum. I must have, in this case, full, free reaction, and I must have it quickly. He will not live until a blister can have its effect! What is to be done? Why, I said to myself, apply the actual cautry. I quickly rubbed his chest with alcohol and applied a lighted taper. The flash of the lurid light had hardly passed off before reaction came on; he became conscious; his breathing is fewer; his pulse fuller and slower; his expectoration easier, etc. I gave him ten grains pulvis doveri and thirty grains sulphur quinine. In half an hour he was breathing much easier and sleeping sweetly. At 10 o'clock, A.M., the next day is almost convalescent; is quite cheerful and comfortable. ℞. sulphate quinine, grs. v.; pulvis dov., grs. iii.; every four hours. Buttermilk, half water, or beef tea at will continued for a few days.

From this time convalescence rapidly supervened. No relapse. I heard lately he is yet enjoying good health in his new home, the State of Missouri.

The month of February, 1855, will long be remembered by me for its warm, damp, heavy, foggy atmosphere. Pneumonia in all its phases was very rife during the whole month, amounting almost to an endemic disease. I was riding day and night, seeing cases principally of pneumonia. On the evening of the 10th, I learned on my return home, from my wife, that in the morning I had received a call to see Mr. Silas C., and that his son John C. was the messenger. I saw from her anxiety to hurry me off she had been impressed with the idea that Mr. Silas C. was dangerously ill. The roads were very bad and I did not reach Mr. C.'s house before 9 o'clock, P.M. When I arrrived at the front door I was surprised, for Mr. S. C., the sick man, opened the door, and the more so, when he excitedly told me he believed John C., his son and the messenger of the morning, was dying. He said John "came home about half past ten o'clock, A.M., looked pale; complained of feeling sick, was sick; raised a little blood; lay down on the bed, and in a few moments was out of his head." I entered the room and found John, a robust young man, aged nineteen, lying in bed on his back; could not be aroused; is comatose; his face is so strongly flushed, that his general appearance bears a strong resemblance to that of a person dead drunk from a poisonous dose of alcoholic stimulants, breathing slow, heavy, laborious; features appear swollen and almost of a purplish color; pulse suppressed, struggling, imparting to the finger a pecular thrill. I see the "brick-dust" sputa on the wall and in the chamber. My first impulse is to open the jugular vein, but a moments reflection convinced me if I did, and the case terminated fatally, as I thought it would before daylight, I knew the unprofessional would say "I cut his throat." I open a large orifice in the vein, but can get no blood, it appears too thick to run; I apply the "cups," the blood will not run, it is black, thick, tarry in appearance; I dip little paper balls in alcohol, set them on fire and scatter them over the chest; this makes him show some signs of life, but very slight. I rub his chest with alcohol, and touched the lighted taper; as the lurid flame flashed over his chest, he raised up in bed, opened his eyes, and took a bewildered survey of the room. I say to him: Lie down, be quiet, you are better now, and he quietly lies down again. His father seeing the little blazing paper balls scattered over his son's chest, the sudden flash of light and hearing the knives of the scarificator grate over the sternum, very modestly asked: "Doctor, what operation do you call that?" "Cupping, I reply." "A pretty rough operation, is it not?" "Yes; but beneficial in extreme cases like the present." In a very few moments he became conscious, and I gave him ten grains hydrargyri chlorid mit; pulvis doveri, grs. x.; castor oil in the morning.

*February* 11.—Bowels have acted well; has slept well; pulse eighty-five, soft; is quiet, natural, comfortable; face pale; expectorates easily; the expectoration is losing its tenaciousness and "brick-dust" appearance. Wants to know if he can go out to work to-morrow. ℞. Pulvis doveri, grs. iii.; sulph., grs. v.; every fourth hour. Buttermilk, half water, or beef tea, *ad libitum.* Dress blister with cloth soaked in linseed oil and molasses. If he is unable to sleep, give pulvis doveri, grs. x., at bed time. Repeat the oil if necessary.

*February* 12.—Is convalescent. After cautioning him to remain in bed for four or five days, to live light, and not to think of leaving the house for the remainder of February, I took my leave. How long he remained in bed I do not know, but on the 19th I had occasion to visit Warrenton. In passing Salem, thirteen miles below, a cold rain was falling. I saw a great crowd in the street, and heard a violin, and some one apparently dancing for the amusement of the crowd. Being naturally of an inquiring turn of mind, I dismounted, hitched my horse, and with difficulty pressed right through the crowd until I came into the presence of the dancer. "*Mirabile dictu!*" who should it prove to be but John C., my patient, so recently laboring under pulmonary apoplexy, cutting the "double shuffle" to a large and an appreciative crowd. He yet lives, and is hale and hearty, by no means injured by his tripping it on the light fantastic toe in the sand to the tune of "Pop Goes the Weasel." His father's was a case of spasmodic colic, and was cured by the scare his son's illness gave him—just as I have before now made a "scare" answer the place of a catheter when I happened to have no catheter with me.

In those cases of pure idiopathic pneumonia, uncontaminated by any malarial influence, I believe aconite—the alcoholic extract of aconite—will be found to be the best remedy—meeting all the benefits to be derived from the lancet, besides procuring sleep and easing pain. In such cases, from my own experience with this remedy, it enjoys my high confidence in its curative properties.

During the winter of 1845, a stout, hearty young man, aged twenty-two, was engaged in attending the medical lectures in the College of Physicians and Surgeons of the State of New York, situated in Crosby street, New York city. He was boarding at 531 Broadway, corner of Spring street. One bright, warm-looking morning in the month of December, he ate a hearty breakfast and started off to attend Dr. Willard Parker's clinic without his overcoat. He was delayed at the crossing by the great number of vehicles passing to and fro. In the meantime, the morning proved deceptive, being very raw, damp and chilly. Before he was able to cross Broadway he was taken ill; he could scarcely get back to the front door of his boarding-house. Not having his night key in his pocket, he was forced to ring the bell for admittance. Charley was slow in answering the bell, and when he did open the door the young man fell, and had to be carried to his room. My friend Dr. Lewis A. Sayre—then a young man, now the distinguished Professor of Orthopedic Surgery in Bellevue Hospital College of New York city—was called to see him. He arrived at 10 A.M.; found him delirious; hot skin; expectorating rust-colored sputa; breathing rapid, painful; complains of acute pain in the chest and side; face flushed; pulse one hundred and twenty, full; violent headache. He gave him five drops extract aconite (homeopathic strength) at ten A.M.; at eleven A.M. the dose was repeated; in ten minutes he was asleep. At six P.M. he awoke, with skin moist, pulse sixty, expectoration easy, free of pain—indeed, entirely relieved. Aconite lowers the circulation, gives ease with sleep, and never runs off by the bowels. Sleep is Nature's best restorative, peculiarly so in pneumonia, for it prevents talking, thereby giving rest to the body, the mind, and to the lungs themselves.

